# Supplementary material for: Community Engagement and Psychometric Methods in Aboriginal and Torres Strait Islander Patient-Reported Outcome Measures and Surveys—A Scoping Review and Critical Analysis
Source: Int J Environ Res Public Health. 2022 Aug 19;19(16):10354. doi: 10.3390/ijerph191610354 (PMC9407920; doi:10.3390/ijerph191610354)
Supplement: Supplementary file 1 [file ijerph-19-10354-s001.zip › Supplementary Material - Table S2.pdf]

Table S2. Scoping Review Psychometric Properties

Process	Property	Definition
Validity	Face	The relevance and readability of the instrument for the target population [15].
	Content	The ability of the instrument in capturing latent variables/main constructs [52].
	Construct	The extent to which the measurement accomplishes the purpose of which it is intended. The relationship between the latent variable and construct of interest [52].
Reliability	Internal consistency	The relatedness of items in instruments to each other in measuring main constructs.
	Repeatability	The ability of instrument scores to be reproduced by same users over a set period where conditions have not changed, though test re-test process.
