# Supplementary figures and images for: Community Engagement and Psychometric Methods in Aboriginal and Torres Strait Islander Patient-Reported Outcome Measures and Surveys—A Scoping Review and Critical Analysis
Source: Int J Environ Res Public Health. 2022 Aug 19;19(16):10354. doi: 10.3390/ijerph191610354 (PMC9407920; doi:10.3390/ijerph191610354)

**Figure S1.** PRISMA flow diagram.

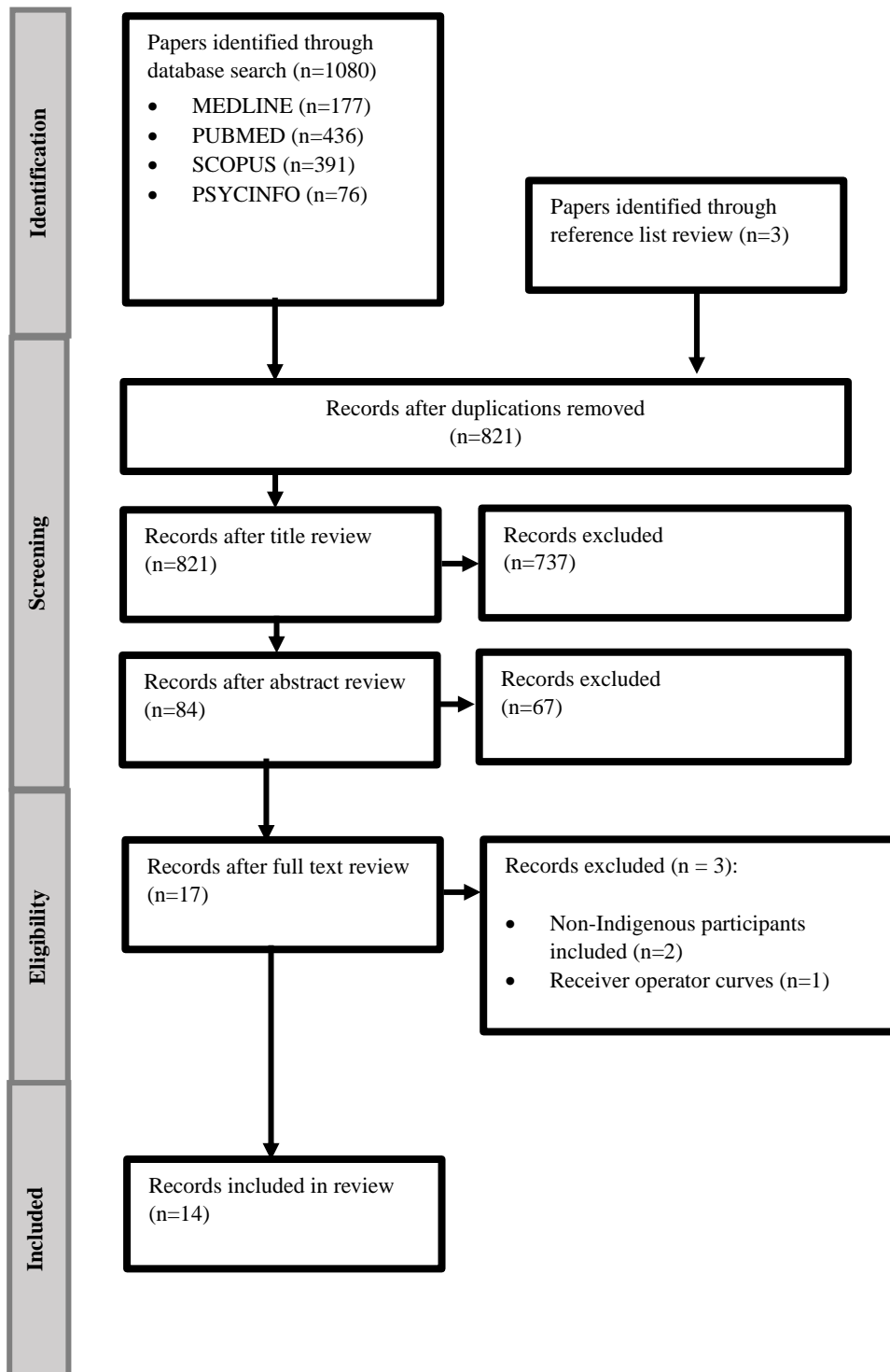

Supplement: Supplementary file 1 [file ijerph-19-10354-s001.zip › Supplementary Material - Figure S1.pdf]
